# Supplementary figures and images for: Long Title: Cas10 based 7SL-sRNA diagnostic for the detection of active trypanosomosis
Source: PLoS Negl Trop Dis. 2025 Mar 17;19(3):e0012937. doi: 10.1371/journal.pntd.0012937 (PMC12051490; doi:10.1371/journal.pntd.0012937)

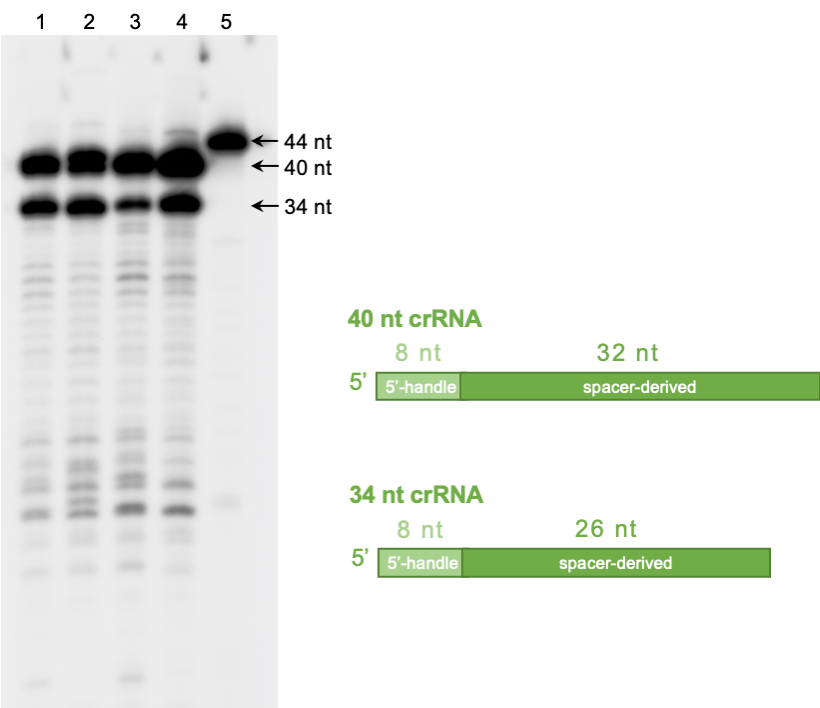

Supplement: S1 Fig — Lane 1: Tco_CA1 crRNA; lane 2: Tco_CA2 crRNA; lanes 3 and 4: crRNA not discussed in the manuscript; lane 5: synthetic RNA oligonucleotide (44 nt). (TIF) [file pntd.0012937.s001.tif]

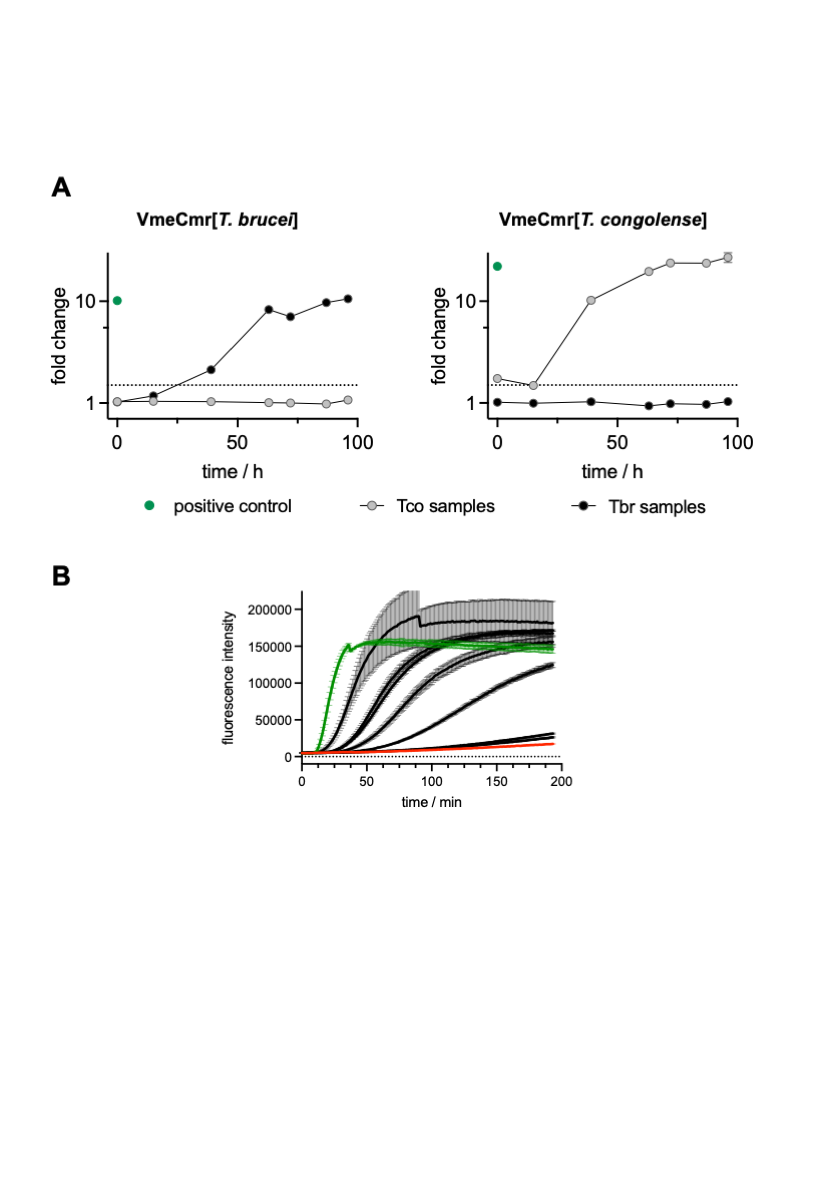

Supplement: S2 Fig — A: Increase in fluorescence after 2 h relative to non-target control (NTC). Samples were analysed with the cognate and non-cognate Cmr complex as indicated. Each replicate is shown with SD. B: Signal curves for the fluorescent assay using the cognate Cmr complex alongside selected synthetic standards to estimate 7SL-sRNA concentration in cell culture samples. Only the mean is shown for clarity. The majority of T. congolense-inoculated samples are above 1 pM and go up to around 10 pM. Surprisingly, these samples did not test positive with the Cmr[Tbr] complex (limit of detection for Tco 7SL-sRNA 1 pM, Fig. 4). This could suggest that specificity is increased rather than decreased in more complex samples. All of the T. brucei-inoculated samples contain 7SL-sRNA below the limit of detection of 1 nM for the non-cognate Cmr[Tco] complex (Fig. 4). (TIF) [file pntd.0012937.s002.tif]

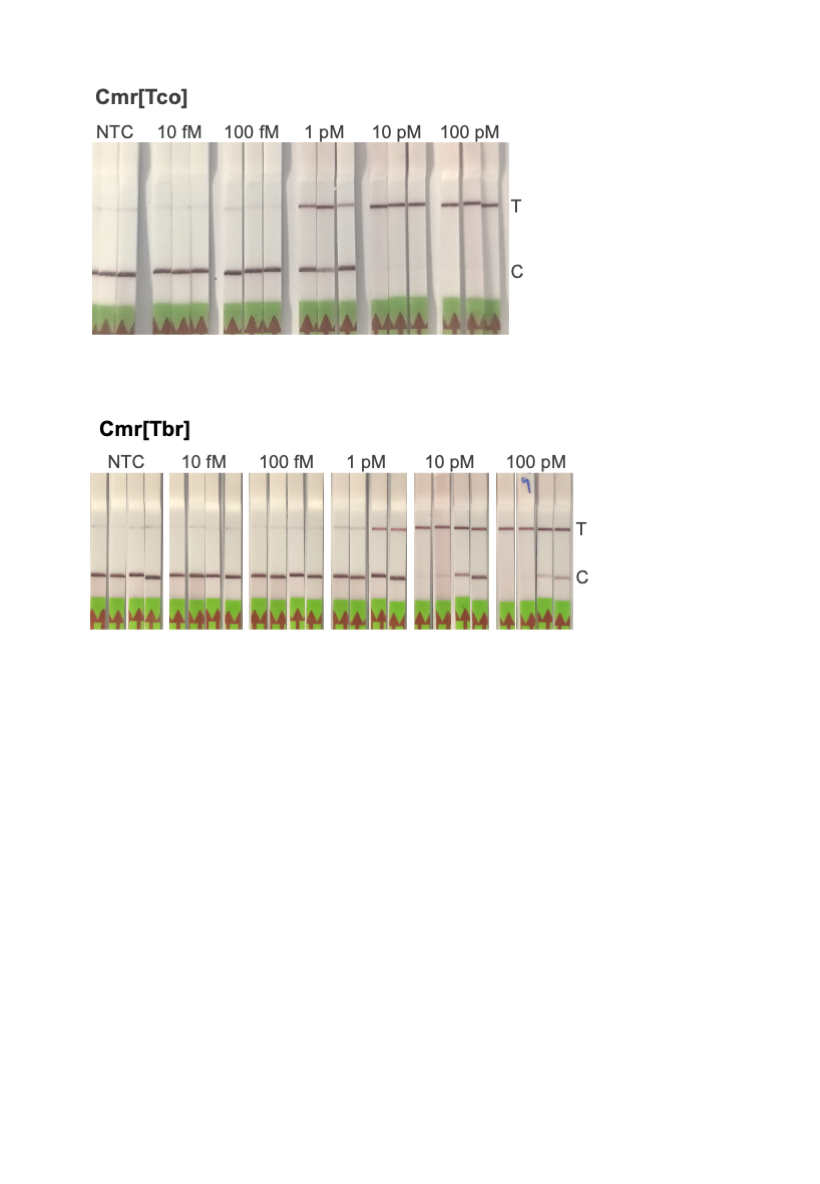

Supplement: S3 Fig — The concentration of the synthetic RNA is indicated above the strips. NTC: non-target control; T: test line; C: control line. (TIF) [file pntd.0012937.s003.tif]

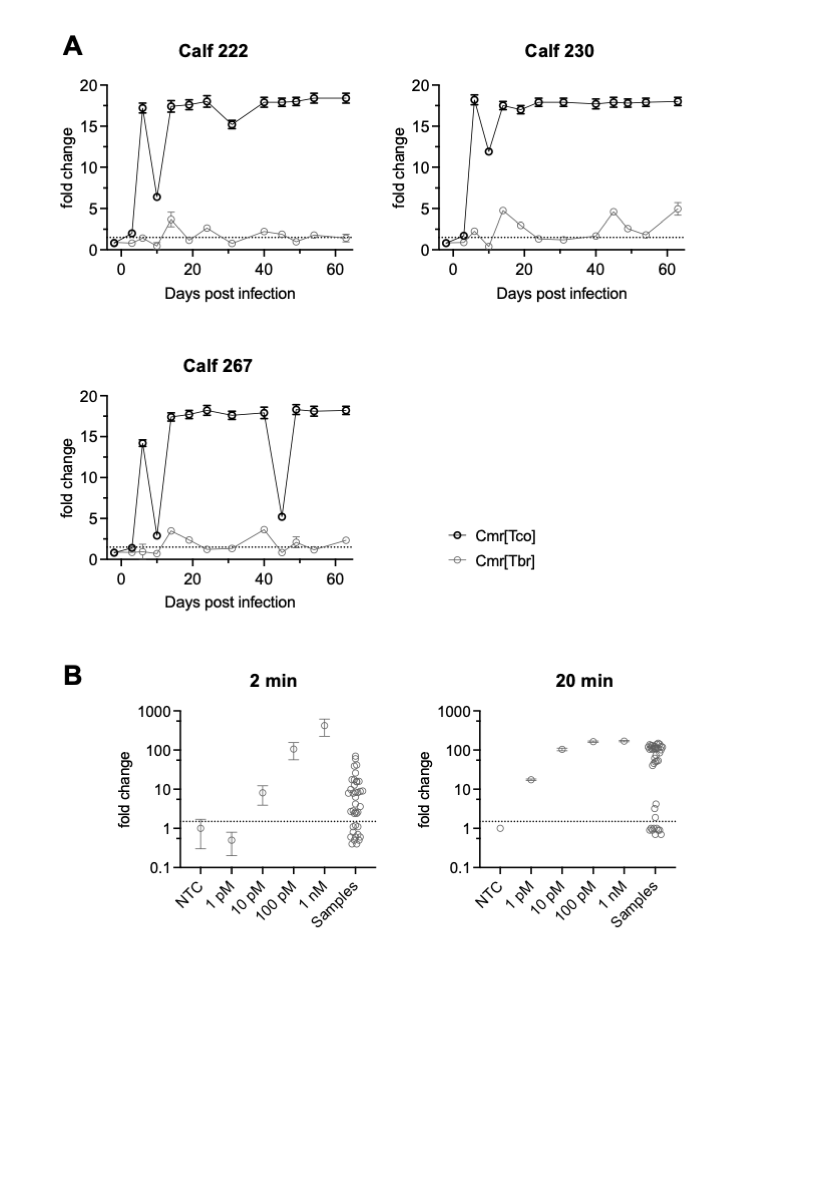

Supplement: S4 Fig — Due to the high concentration of Tco7SL-sRNA in these samples (up to between 10 – 100 pM as judged by comparison to synthetic standards as shown in B), a high number of samples tested positive with the non-cognate Cmr[Tbr] complex. The reaction time was 2 h in A, and 2 and 20 min in B as indicated above the graph. Mean values plus SEM are shown except for the samples in B where only the mean fold change is plotted. (TIF) [file pntd.0012937.s004.tif]
