# Supplementary material for: Long Title: Cas10 based 7SL-sRNA diagnostic for the detection of active trypanosomosis
Source: PLoS Negl Trop Dis. 2025 Mar 17;19(3):e0012937. doi: 10.1371/journal.pntd.0012937 (PMC12051490; doi:10.1371/journal.pntd.0012937)
Supplement: Attachment — Submitted filename: Reviewers Comments_R1.docx [file pntd.0012937.s007.docx]

We thank the reviewers for their time and constructive comments. We have addressed each comment either through additional experiments or clarification in the text of the manuscript. We believe the changes made, as suggested by the reviewers, has strengthened the manuscript.

**REVIEWER COMMENTS**

***Comment:*** *The use of words “trypanosomiasis” in the title text (line 2 & 3) and “trypanosomosis” in the body of the text (line 20, line 38, line 57 etc…) may be confusing to non-specialist readers. I would advise the authors to adopt any one of the two words for the entire text as appropriate.*

**Response:** Thank you for pointing this out. We have made the changes as suggested, and have used ‘trypanosomosis’ to align with FAO and WOAH recommended nomenclature.

***Comment:*** *Line 117. Please revise the sentence to bring 7SL-sRNA “inside” the sentence.*

**Response:** We have changed the text to “Therefore, 7SL-sRNA ......”

***Comment:*** *Line 126. Define abbreviation cA3 in Fig 1 legend.*

**Response:** This has now been defined

***Comment:*** *Line 205. The word “each” carries a capital “E”. Should be changed to a small letter “e”.*

*Line 231. The sentence: “…transferred to a new sterile tube…”. The word “to” should be replaced with “into”.*

*Line 271. The sentence reading “The second, Tco_CA2 could…”.  Please, insert a comma immediately after CA2.*

*Line 215. To ensure a logical flow of information in the Materials and Methods, I would advise that authors to describe RNA extraction first followed by qPCR and then end with LFA.*

**Response:** We can confirm that all above comments have been addressed as advised.

**C*omment:*** *Line 276. Increase the resolution of Figures 2, 4, 5, and Fig S4.*

**Response:** Low resolution images were included for initial review. We will ensure high resolution images will be included in the final manuscript.

***Comment:*** *Line 293 and 294 (Fig 3). For purpose of clarity, the authors should distinctly label the two graphs. Graph on the left side could be assigned letter “A”, and right-hand “B”. Could the author provide explanation why the curves for 100 pM to 10 nM are missing from the graph of “fold change vs time” (right hand-side)?*

**Response:** Thank you for pointing this out. That was a mistake on our part. Curves for 100 pM to 10 nM have been included in the right-hand figure.

***Comment:*** *Line 297. The “non-target control” should be specified.*

**Response:** We have now added a non-target RNA sequence to Table 1 and changed control RNA to non-target RNA (Table 1).

***Comment:*** *Line 309-Line 310 (Figure 4). The graphs should be made distinct from the aligned sequences.  For example, assign the graphs as “A” with sub-levels (i-iv) and the aligned sequences as B. Thereafter, in each case, make a proper description of the figures in the legend.*

**Response:** Fig. 4 has been changed as suggested. We now include three independent experiments for each combination of Cmr complex and target RNA, while the legend has been changed as follows:

“including cross-species specificity” has been changed to “and discrimination against related species. A:”

“SEM” has been changed to “SD”

“The pairwise alignment of the two 7SL-sRNA species used in this study is shown below the graphs.” has been changed to “B: Pairwise alignment of the two 7SL-sRNA species used in this study.”

“for cognate RNA, and from technical duplicates for non-cognate RNA. PC: positive control (10 pM cognate 7SL-sRNA, green);” has been removed.

***Comment:*** *Line 360. It reads “Fig 6B and C shows the LFT results…”. It should read “Fig 6C and D”, rather, not B. Fig 6B are illustrations meant for interpretation of the assay's results.*

***Comment:*** *Line 377. C, D should be written as C and D.*

**Response:** The text has been changed to address both comments.

***Comment:*** *Line 386. Application of microscopy for examination of sample should be mentioned/brought earlier in the Materials and Methods or, at least, be referenced. I only encountered it in the results section.*

**Response:** Apologies for this oversight. We have added in methodology for parasitaemia (in vivo) and cell density (in vitro) measurements in the relevant sections of the Materials and Methods.

***Comment:*** *Line 479 - 484. I am not in agreement with the statement made by the authors that DNA and conventional RNA detection techniques require extraction of these biomarkers from intact trypanosomes yet the “new” 7SL-sRNA biomarker is released in the sample and, therefore, can be detected by their newly developed tool even in the absence of Trypanosoma cells. Unless experimentally proven, the fact that they had to extract RNA prior to detecting 7SL-sRNA may mean this biomarker is located intracellularly and only released in the milieu by decomposing cells. In my opinion DNA or any other forms of RNA are often released by decomposing Trypanosoma cells. The statement indicating that 7SL-sRNA can be detected without the need for intact parasite may be subjective. You may make this clear by answering the following questions: i) How long does the 7SL-sRNA biomarker remains detectable after clearing trypanosomes by drug treatment or after self-cure? ii) What mechanism does*
*trypanosome use to excrete/secrete 7SL-sRNA?*

**Response:** We have previously demonstrated that during the exponential growth phase during in vitro culture, when there is limited death of parasites, we consistently detect increasing levels of 7SL-sRNA, at far higher levels in RNA extracted from the supernatant compared to RNA extracted from the parasite cells in the same cultures – these observations are inconsistent with release from dead/dying cells (Chiweshe et al., 2019). Furthermore, we were able to show that we could detect 7SL-sRNA directly from serum using the RT-qPCR assay, without prior RNA extraction. We also showed that 7SL-sRNA could be detected in the serum of infected cattle when no parasites were detected in the blood. Small RNA cloning and sequencing from serum and supernatant from in vitro cultures also showed 7SL-sRNA present at levels far higher than that of any other parasite derived RNA. These data collectively strongly support active secretion or excretion of 7SL-sRNA, rather than a passive mechanism such as release following death of parasites. If this were the case, we would expect to see equally high levels in the parasite and evidence of other parasite derived RNAs being released during decomposition of parasites, and it is very unlikely we would detect such a difference in 7SL-sRNA levels between supernatant/serum and the parasite cells. The signal rapidly declined once the cattle were treated with anti-trypanosome drugs indicating viable parasites were required to maintain 7SL-sRNA signal in the blood, even during lag phases of infection. While we have not formally demonstrated direct detection of 7SL-sRNA from serum samples with the Cas10 assay, the ability to do so with the RT-qPCR assay demonstrates the feasibility of this approach. Studies are ongoing to understand the precise mechanism by which 7SL-sRNA is excreted/secreted.

***Comment:*** *Line 509. What are the levels of sequence similarity between the 7SL-sRNA of T. brucei or T. congolense and other pathogenic species of animal trypanosomes when compared to T. theileri? Could the sequence divergence offer explanation why the tests did not cross-react with T. theileri?*

**Response:** In a previous study, we compared the 7SL-sRNA sequence of several trypanosome species, including T. theileri (Contreras Garcia et al., 2022, PMID: 35450136). Here, we showed that the RT-qPCR assays for T. congolense and T. brucei (as well as T. vivax) exhibited no cross reactivity with T. theileri. This is referenced in the introduction. Although we have not formally shown that the Cas10 assay does not cross-react with T. theileri, given the sequence differences between T. brucei/T. congolense and T. theileri shown in this previous study, and the lack of cross-reaction with the 7SL-sRNA qPCR assays, we consider this highly unlikely.

**Reviewer #2:**

***Comment:*** *Along the entire manuscript, human African trypanosomiases and animal African trypanosomiases are sometimes mixed, which is confusing. Please, carefully distinguish the human and animal diseases, homogenize the terminology, and clearly state that this work is focusing on T. congolense and T. brucei sl. Only.*

**Response:** Thank you for pointing this out. This has now been corrected.

***Comment:*** *There are no statistical analyses of the results. The variability of the results, that should be presented as SD not SEM, is apparently not shown in all figures. Were all tests performed at least in triplicates? These aspects should be improved.*

**Response:** For design and validation of the assays, synthetic RNAs are used with precise concentrations known. Because of this there is very little variance between technical replicates, making additional technical duplicates redundant. This can be seen in figures 2 and 3. However, we have now repeated the cell culture infection time course and sampled RNA in triplicate. The results from the repeat experiment replaces the existing results in Figures 5 and S2. The legend texts for both Figures have been changed accordingly.

The animal study encompasses three animals, i.e. biological triplicates, and shows the natural variation of infection progress within the different animals. SEMs have been changed to SDs in Figures 2, 4, 5 and S4. The error bars in Fig 7, fluorescence fold change are too small to be visible but are in Fig S4.

***Comment:*** *This work presents a very promising test, especially because it doesn’t require any pre-amplification step, yet the results could be discussed more cautiously. Comparison to other molecular tests should remain fair and the high level of criticism imposed to the other methods should also be applied to the Cas10 based 7SL-sRNA assay.*

**Response:** We agree and have adjusted the text accordingly.

***Comment:*** *The investigations are focusing to 2 trypanosome species only, with a limited specificity (Fig.4). The observed cross-reactivity between assays is not minimal: in the case of a mono-infection with detectable parasitemia, both Cas10 based 7SL-sRNA tests on blood will likely be positive for both species. Moreover, it would be important to further assess the specificity of the Cas10 based 7SL-sRNA assays by testing other trypanosome species, other sympatric parasites, different sample types, with host genetic material… - The lower LoD is apparently good, yet around 1pM in most figures, not 100fM. For easier comparisons, the use of the standard parasites/ml as reference unit would be appreciated.*

**Response:** The LoD for Cmr[Tco] and Cmr[Tbr] against their cognate targets was determined to be 10 - 100 fM and 1 pM, respectively. There is cross-reactivity with the Cmr[Tbr] complex as stated in the manuscript, however, the Cmr[Tco] complex shows good discrimination against the related T. brucei target. We do show what the specificity and lack thereof looks like with clinically relevant samples. It is true that testing a wider range of trypanosome species will be formally necessary. However, as this is the first proof-of-principle study, we feel that this is beyond the scope of the paper and instead, this is something we will address in future studies. As mentioned by Reviewer 3, the cross-reactivity between *T. brucei* and *T. congolense* is not necessarily a disadvantage as the treatment would be the same for both species.

It is very challenging to relate 7SL-sRNA abundance to “parasites/mL” as RNA expression is dynamic, and individual parasites may generate variable amounts of this particular RNA species. In addition, the 7SL-sRNA we are detecting is extracellular. Whilst 7SL-sRNA abundance does appear to correlate to cell density under *in vitro* conditions, this is clearly not the case during *in vivo* infections, as abundance remains high during chronic infection, when parasitaemia is undetectable via microscopy (in addition, 7SL-sRNA is quite stable in culture, and abundance remains high past peak cell densities, as can be seen in Figure 5B - this makes correlations to parasites/mL difficult). The closest reference standard we could relate molarity to is mass of RNA molecule per mL. For example, one could calculate that an abundance of 1 pM of the T. brucei 7SLs-RNA is equivalent to 7.83 pg/mL. However, we have not experimentally verified this, and in addition, we do not believe mass per mL would carry any advantage compared to molarity, and we have therefore elected to use the latter.

***Comment:*** *As mentioned in the discussion only, the assay is not already adapted to the field because an RNA extraction is required prior to the Cas10 assay and the commercial LFA is not adapted for field use (lack of real control band).*

*In total, along the entire manuscript, it would be greatly appreciated to tone down a bit the enthusiastic promotion of this new assay that is indeed promising, but that still requires a significant work of assessment and improvement before to be usable in the field. According to the data presented in this article, it is difficult to agree that the currently proposed Cas10 based 7SL-sRNA detection assays are highly sensitive and specific.*

**Response:** We appreciate the Reviewer’s comments, and we have adjusted the text accordingly.

***Comment:*** *The title doesn't seem to be correct. First, the authors present a Cas10 based 7SL-sRNA detection assay for the diagnostic of active trypanosomiasis. Second, as only T. congolense and T. brucei sl. were investigated, the word ‘trypanosomiasis’ should be clarified.*

**Response:** We have updated the text to trypanosomosis. However, as the assay does detect more than one causative agent of trypanosomosis, the title is correct in this sense, and we do not believe this is misleading.

***Comment:*** *L30. The LFA used in this study is a commercial one, hence it was not developed by the authors.*

**Response:** We do not agree with this statement. While the strips are a commercial product (as a plate reader or qPCR machine would be), we designed and developed the combination of probe and enzymatic reagents that were applied to the strip to develop the LFT assay, and we therefore maintain that the assay was developed by us.

***Comment:*** *Please, clearly present the analytic sensitivity and specificity of the assays in the abstract with numbers.*

**Response:** “we have developed a lateral flow diagnostic test” now reads “we have developed both a fluorescence-based and a lateral flow diagnostic test”

“This assay demonstrates high sensitivity and specificity, effectively identifying active infections in cattle” now reads “The fluorescence assay detects 10 – 100 fM T. congolense 7SL-sRNA and 1 pM T. brucei 7SL-sRNA, and the lateral flow assay showed a limit of detection of 1 – 10 pM for both species. Either assay is able to effectively identify active infections in cattle”.

***Comment:*** *Please, clearly indicate that only T. congolense and T. brucei sl. were investigated.*

**Response:** “Cas10 technology for the detection of 7SL-sRNA.” now reads “Cas10 technology for the detection of 7SL-sRNA from Trypanosoma congolense and T. brucei”

***Comment:*** *L59. T. vivax can also be transmitted after cyclical development in the tsetse mouthparts.*

**Response:** Apologies, this was an oversight on our part and has now been corrected.

***Comment:*** *L63: according to the WHO reference cited, Tbg is responsible for 92% of reported cases and Tbr for 8%.*

**Response:** Apologies for this error. This has now been corrected.

***Comment:*** *L84-88: The list of diagnostic assays presented in this paragraph does not reflect the current diagnostic algorithm recommended by WHO. Please clearly distinguish the tests that are available and routinely used in the recommended diagnostic algorithm from the additional methods used for research, surveillance and epidemiological studies.*

**Response:** This has now been clarified, and separated into microscopy of blood, CATT test and microscopy of CSF following lumbar puncture (as recommended by WHO), and PCR tests to detect parasite DNA that have also been developed, but which do not currently form part of the formal diagnostic cascade.

***Comment:*** *L107: More than 3 trypanosome species can infect animals.*

**Response:** This has been amended to read: ‘from the three species of African trypanosome responsible for the majority of AT and HAT cases (*T. congolense*, *T. vivax* and *T. brucei*).’

***Comment:*** *L135-137: SHERLOCK is detecting RNA alone (or both RNA and DNA according to the strategy), but not DNA alone. Please correct this misleading statement.*

**Response:** The line: “However, both of these approaches are DNA based, and so rely on pathogen DNA being present in the target sample, which is often a limitation in chronic trypanosome infections where parasite numbers are very low.” - has been deleted.

***Comment:*** *L146: A femtomolar detection level is observed for one assay in one condition shown in one figure. Hence, this statement could possibly be toned down.*

**Response:** The text has been adjusted accordingly.

***Comment:*** *Fig 5 is difficult to read. Could you please propose an alternative simpler representation? Could you use a simpler scale for the parasitemia? Could you especially highlight at what parasitemia the Cas10 assays start detecting parasites?*

**Response:** We have changed the presentation of the data in Fig 5 as detailed in the amended legend. Original legend to Fig 5 has been changed to: “Fig 5. 7SL-sRNA detection from in vitro T. congolense (A) and T. brucei (B) cell culture supernatants. The graphs contain results from the Cmr/NucC fluorescence and RT-qPCR assays (left *y*-axis) as well as the parasite cell count (right *y*-axis). Filled symbols indicate a positive test (above threshold), whereas open symbols indicate a negative test result for the Cmr / NucC and qPCR assays. The mean and SD from triplicates are shown.”

The parasitaemia scale is what would be familiar to most readers working on trypanosome biology (parasites/ml), and therefore we are reluctant to change this. We also believe it is evident from the graph that the assay is negative (y axis) at time 0, but positive thereafter (i.e. from first timepoint at 24 hours). Because of the intermittent sampling we not think it appropriate to highlight a point at which the assay first gives a signal (in any case this is 24 hours in all cases), as this could result in a misleading conclusion re thresholds.

We have also changed the representation in Fig 7 in the same way as for Fig 5 to maintain consistency. The legend has been amended to reflect the changes.

As the representation of the qPCR results has changed, we added the following statement to the qPCR section in Materials and Methods: “Results from qPCR are expressed as the difference in Ct value to the negative control (Ct = 40) for ease of representation.”

***Comment:*** *In Fig 5 and 6, how do you explain the long delay before to observe positive results in the Cas10 assays?*

**Response:** Thank you for pointing this out. There was an error in the x-axis that referred to days instead of hours. This has now been corrected.

***Comment:*** *From Fig 7, qPCR and Cas10 assay show similar results. In the text, it is mentioned that 4 false negative results were obtained in the Cas10 assay. How many in qPCR?*

**Response:** There were no false negatives for qPCR in this experiment.

***Comment:*** *Do you have any idea of the stability of the excreted/secreted sRNAs in blood? How long could a 7SL-sRNA molecule remain intact and detectable in animal blood?*

**Response:** In a previous study led by collaborators, it was found that the 7SL-sRNA remains detectable in positive sera after 7 days of storage at either 4°C, room temperature, or 30°C (Verney et al., 2020; PMID: 32687668). It is therefore very stable in blood samples. We are less sure about the stability in blood in living host. For example, in treated animals, the signal drops rapidly (24-28 hours) post-treatment (Contreras Garcia et al., 2022: PMID: 35450136), suggesting that live trypanosomes are required to sustain 7SL-sRNA generation and that the molecules are rapidly destroyed by the host. Determining the precise stability of 7SL-sRNA *in vivo* is very challenging, as multiple factors determine the length of time a signal can be detected, including how quickly drugs affects parasite numbers and the potential for loss of signal due to clearance during circulation of blood supply.

**Reviewer #3:**

***Comment:*** *Given that the treatment options for T. brucei and T. congolense overlap (as far as I am aware)– why is it important to discriminate between the two for treatment? Would it not be better to be able to discriminate between T. brucei/T. congolense and T. vivax?*

**Response:** We acknowledge that a universal assay would be very useful in this context – this will be the focus of future work. However, there are reasons as to why species-specific assays could have significant utility. Firstly, the curative dose for the most widely used trypanocide is approximately double for T. brucei compared to the dose for T. congolense, meaning accurate diagnosis could lead to both more appropriate drug use and less risk of under-dosing (with consequent risks for resistance selection). Secondly, only variants of T. brucei cause human disease. Currently DNA PCRs for T. brucei are often used as a first pass test in molecular surveillance (due to high sensitivity), and the PCRs for human-infectivity (which are more complex and involve two targets and a nested PCR protocol) are then applied to the T. brucei positive samples. Therefore, in this context a sensitive assay that detects active T. brucei infection could indeed be highly useful.

I would expect the discrimination of Cmr[Tco] and Cmr[Tbr] against vivax to be better than Cmr[Tco] against Tbr or Cmr[Tbr] against Tco as mismatches between Tbr and Tco start after 10 nt into the crRNA base-pairing region whereas for Cmr[Tco/Tbr] against vivax mismatches start after 4 nt (segment 1). These “early” base-pairings, ie those closest to the PFS tend to have a bigger impact on activation of the Cas10 cyclase domain (but would need to be verified in our system as unexpected behaviours do occur).

***Comment:*** *It is stated several times that Cas10 would be a cost-effective diagnostic, could you please include a prospective costing of both a high-throughput assay and RDT in comparison to what is currently available.*

**Response:** We are not at the stage of being able to provide an estimate of cost – given this will be reliant on final product and scale of production, we do not believe it will be helpful to provide a figure that may be ultimately misleading. However, given the enormous acceleration of LFT technology innovation following the COVID-19 pandemic, we do believe that it is reasonable to say that assays based on these platforms will be cost-effective going forward.

***Comment:*** *The benefit of this diagnostic is that, by detecting extracellular 7SL you overcome low parasite number. But would you not expect the levels of extracellular 7SL to fluctuate with the levels of parasitemia and/or be degraded over time? There seems to be some indication of this in Fig 5, where there is a slight increase in the fluorescence over time. However, in Figure 7 the fluorescent signal appears to plateau even though parasiteamia fluctuates. Could you explain why this is the case and why you anticipate long term preservation of extracellular 7SL RNA in the serum.*

**Response:** As shown here and in Chiweshe et al. 2019, the signal for 7SL-sRNA remains detectable even when parasitaemia cannot be detected. We hypothesise that during this period, parasites within tissues continue to generate 7SL-sRNA which can be detected in the blood of infected animals – however, this cannot be formally proven without substantial experimental work. We do however see a broad correlation with parasite numbers as the signal does begin to drop during prolonged periods of latent infection.

***Comment:*** *Fluorescent 7SL-sRNA assays need to be done in triplicate and at least the SEM reported, best with the three data points plotted for each time point. Figure 2 states mean of technical duplicates; Figure 4 states SEM from 3 independent experiments, and Figures 5 and 7 read as though this is a single experiment. How reproducible is the assay, especially in serum from the cattle experiment? If it is only one assay in Figure 5 and 7 these should be repeated to at least generate triplicate data that at least error bars can be given for.*

**Response:** Please see the previous response to reviewer 2. Additional repeats of the cell culture experiments have now been included.

***Comment:*** *Can you give the analytical sensitivity and specificity of both the Tb and Tc assays with 95 % CI.*

**Response:** To ascertain accurate analytical sensitivity and specificity measurements for these assays, we would require a large number of samples to work with, which is outside the scope of this study. We agree with the Reviewer that these values are important to generate and it is a future aim. We have alluded to this in the discussion section.

***Comment:*** *Many infections in a natural setting are most likely to be co-infections between 2 or more species. Currently, none of your assays tests the diagnostic where species are mixed. For in in vitro assay, you should do a mixing experiment of a series of proportions between Tb and Tc (ie 1:1 ; 1:5; 1:10 – 5:1; 10:1) and determine the specificity of each Cas10 assay.*

**Response:** This was an interesting question posed by the Reviewer. Based on what we know about the limits of detection of the individual Cas10 complexes towards their cognate and cross-species targets, we assume these limits would not differ, even under “mixed infection” conditions. To illustrate this, we carried out a single experiment where we mixed the T. brucei and T. congolense synthetic 7SL-sRNA at varying proportions:

Total synthetic 7SL-sRNA was kept to 10 pM by mixing different ratios of Tco and Tbr standards as indicated. The samples were then analysed via the Cas10 fluorescence assay. As expected, even under mixed conditions, the assays still perform according to the calculated limit of detection of the individual complexes towards their cognate and the cross-species targets. That is to say, Cmr[Tco] only reacts to the Tco target (left) whereas Cmr[Tbr] reacts also to Tco targets above > 1 pM (right; orange data points indicate controls where only Tbr 7SL-sRNA was used).

***Comment:*** *1) Line 57 add (T) after Trypanosoma; 2) Line 59 – one bracket; 3) Line 134 – 135 Cas13a is RNA detecting not DNA: see - Abudayyeh, O. O., et al. RNA targeting with CRISPR-Cas13; Nature 2017 Vol. 550 Issue 7675*

**Response:** We thank the reviewer for raising these errors. We have addressed 2) and 3). However, we were not sure what the Reviewer was referring to with 1)? It is not common nomenclature to add an abbreviated genus name in brackets after the full genus name.
